# *Trans*‐acting translational regulatory RNA binding proteins

**DOI:** 10.1002/wrna.1465

**Published:** 2018-01-17

**Authors:** Robert F. Harvey, Tom S. Smith, Thomas Mulroney, Rayner M. L. Queiroz, Mariavittoria Pizzinga, Veronica Dezi, Eneko Villenueva, Manasa Ramakrishna, Kathryn S. Lilley, Anne E. Willis

**Affiliations:** ^1^ MRC Toxicology Unit Leicester UK; ^2^ Cambridge Centre for Proteomics, Department of Biochemistry University of Cambridge Cambridge UK

**Keywords:** interactome capture, IRES uORF, mRNA translation, protein synthesis, RNA binding protein, terminal oligopyrimidine tract

## Abstract

The canonical molecular machinery required for global mRNA translation and its control has been well defined, with distinct sets of proteins involved in the processes of translation initiation, elongation and termination. Additionally, noncanonical, *trans*‐acting regulatory RNA‐binding proteins (RBPs) are necessary to provide mRNA‐specific translation, and these interact with 5′ and 3′ untranslated regions and coding regions of mRNA to regulate ribosome recruitment and transit. Recently it has also been demonstrated that *trans*‐acting ribosomal proteins direct the translation of specific mRNAs. Importantly, it has been shown that subsets of RBPs often work in concert, forming distinct regulatory complexes upon different cellular perturbation, creating an RBP combinatorial code, which through the translation of specific subsets of mRNAs, dictate cell fate. With the development of new methodologies, a plethora of novel RNA binding proteins have recently been identified, although the function of many of these proteins within mRNA translation is unknown. In this review we will discuss these methodologies and their shortcomings when applied to the study of translation, which need to be addressed to enable a better understanding of *trans*‐acting translational regulatory proteins. Moreover, we discuss the protein domains that are responsible for RNA binding as well as the RNA motifs to which they bind, and the role of *trans‐*acting ribosomal proteins in directing the translation of specific mRNAs.

This article is categorized under:

1 RNA Interactions with Proteins and Other Molecules > RNA–Protein Complexes
2 Translation > Translation Regulation
3 Translation > Translation Mechanisms

## 1 INTRODUCTION

The process of cap‐dependent translation can be divided into three central events: initiation, where eukaryotic initiation factors (eIFs) assemble an elongation competent ribosome at the start codon (Hinnebusch, 2014); elongation, where the ribosome and eukaryotic elongation factors (eEFs) decode the mRNA into a polypeptide (Hershey, 1991); termination, in which the ribosome disassembles after encountering a stop codon and releases the nascent polypeptide, a process that is aided by eukaryotic release factors (eRFs; Jackson, Hellen, & Pestova, 2012). Protein synthesis is regulated by controlling the bioavailability of eIFs and eEFs through phosphorylation (Hinnebusch, 2014) and in some cases, their cleavage (Bushell et al., 2006). In general, such changes to the canonical machinery occur under conditions of pathophysiological stress including viral infection, changes in ambient temperatures, cell starvation and hypoxia; allowing reprogramming of the translatome to direct cell fate (Spriggs, Bushell, & Willis, 2010). Under these conditions initiation can be decreased via the phosphorylation of eIF2, which reduces the availability of ternary complex (TC; comprised of eIF2, tRNAi^Met^ and GTP); and dephosphorylation of 4E‐BP, which enhances its binding to the cap‐binding protein eIF4E, inhibiting eIF4F formation (which consists of the scaffold protein eIF4G, the dead box helicase eIF4A, in addition to eIF4E). Recent data has also highlighted the importance of translational control exerted via the regulation of elongation (Richter & Coller, 2015) and this is achieved, in part, through changes in the phosphorylation status of eEF and cellular tRNA composition under a range of conditions, including nutrient deprivation (Leprivier et al., 2013), cell differentiation (Gingold et al., 2014), and cold stress (Bastide et al., 2017). In addition to control by the canonical translational machinery (Hinnebusch, 2014), numerous RNA binding proteins (RBPs) behave as *trans*‐acting regulatory factors, which either repress or stimulate the translation of specific subsets of mRNAs. It has been shown that individual RBPs are able to coordinate the translation or repression of specific mRNAs by interacting with defined motifs/structural elements in 5′ and 3′ untranslated regions (UTRs), or coding regions, and thus direct cytoplasmic gene expression programs that are required to respond to changes in external conditions. Gaining a complete understanding of cytoplasmic control of gene expression is further complicated by the fact that a combinatorial code exists for RBPs, where complexes of different subsets of RBPs are formed under distinct cellular conditions to direct specific cellular processes, such as apoptosis (King et al., 2014). In addition, recent studies have shown that the number of RBPs is much greater than originally proposed (Castello et al., 2012). In this review we will discuss methods which have been devised to identify novel RBPs, protein domains associated with RNA‐binding, and RNA motifs/elements which are bound by RBPs. In particular, we describe four representative RBPs that stimulate and repress the translation of subsets of mRNAs, and discuss the role of *trans‐*acting ribosomal proteins in directing the translation of specific mRNAs.

## 2 METHODS TO IDENTIFY AND CHARACTERISE RNA BINDING PROTEINS

Techniques for the identification of RBPs may be broadly split into two categories: (a) RNA‐centric, including RNA‐binding proteome (RBPome) capture and RNA affinity methods to identify proteins binding cellular RNA; and (b) protein‐centric methods, such as RIP and CLIP, to establish all RNAs bound to a single protein. We refer the reader to the following reviews for more complete discussions of the techniques involved in RNA‐centric and protein‐centric identification of RNA binding sites (Faoro & Ataide, 2014; Marchese, de Groot, Lorenzo Gotor, Livi, & Tartaglia, 2016), as well as methodologies that can be employed for the in vitro identification of RBPs (Campbell & Wickens, 2015).

To comprehensively identify the RBPome, a combination of ultraviolet (UV) crosslinking and oligo(dT) purification (RBP‐capture) has been used (Baltz et al., 2012; Castello et al., 2012; Mitchell, Jain, She, & Parker, 2012). UV irradiation at 254 nm generates short lived (μs) radicals that react with amino acids at “zero distance” to form a covalent bond with the nucleotide base of the RNA, thus fixing direct protein–RNA interactions (Pashev, Dimitrov, & Angelov, 1991). Similarly, UV irradiation at 365 nm crosslinks photoactivatable nucleoside analogues, 6‐thioguanosine (6‐SG) and 4‐thiouridine (4‐SU), to amino acids (PAR‐CL). It has been suggested that both 254 nm and 365 nm wavelengths can be used since the chemistries are distinct (Castello et al., 2012), however most RBP‐capture methods use just one wavelength (Baltz et al., 2012; Mitchell et al., 2012). Following crosslinking, RNA‐bound proteins are purified using oligo(dT) beads and identified via mass spectrometry (MS). Controls are necessary to aid removal of false positives. This is ideally achieved through alternative labeling of UV‐positive and negative samples through stable isotope labeling with amino acids in cell culture (SILAC), so the enrichment of the protein following crosslinking can be quantified, from which a threshold can be established for the minimal enrichment required to define a protein as RNA binding. The alternative subtractive approach relies solely on protein identification and involves exclusion of all proteins identified in the control sample (minus UV exposure). There are some limitations to RBP‐capture, as noted by the authors (Castello, Horos, et al., 2013); the protocol requires large amounts of starting material (approximately 10^8^ cells) and the use of oligo(dT) can bias the purification to that of polyadenylated RNA. In addition, because crosslinking only occurs between amino acids and RNA bases in direct contact, RBPs that interact with the sugar phosphate backbone or double stranded RNAs, or indirectly via a protein complex, may not be efficiently crosslinked. However, the key benefit of RBP‐capture is the use of stringent washes to remove contaminating proteins (noncovalently bound proteins and RBP‐interacting proteins), and rigorous bioinformatic analysis, to provide a list of RBPs with high‐confidence. Accordingly, this technique has become the standard approach to identify the RBPome and has been applied to multiple species including *Leishmania donovani* (Nandan et al., 2017), *Saccharomyces cerevisiae* (Mitchell et al., 2012), *Arabidopsis thaliana* (Marondedze, Thomas, Serrano, Lilley, & Gehring, 2016), *Drosophila* (Sysoev et al., 2016), and mammalian systems such as mouse embryonic stem cells (mESCs; Kwon et al., 2013) and human cell lines including HeLa (Castello et al., 2012), and HEK293 (Baltz et al., 2012).

A wide range of RNA‐centric methods exist that use RNAs as a bait to capture bound proteins, which are then identified by MS. Most methods rely upon a high affinity interaction between the bait RNA and a protein, peptide or other small molecule, to either immobilize the RNA on a solid phase from which the RNA–protein interactions of interest can be formed in vitro, or to purify the protein‐bound RNA from cell lysate in which the RNA–protein interactions have been formed in vivo. In addition to the in vitro/in vivo partition, the other major difference between the methods is whether the protein interactions detected are with the native RNA or a tagged RNA. Tagged RNA‐based methods exploit high affinity binding to chemical tags incorporated during in vitro RNA synthesis, or oligonucleotide sequences (aptamers) incorporated during in vivo or in vitro synthesis. For example, streptavadin has frequently been used in combination with either biotinylated nucleotides (Sharma, 2008) or streptavidin aptamers (Srisawat & Engelke, 2001), due to the high affinity of these interactions. Another commonly used high affinity protein‐aptamer combination is the bacteriophage MS2 coat protein and its cognate RNA (Bardwell & Wickens, 1991). The drawback of both chemical modification and aptamer incorporation is that they may alter the secondary structure of the RNA and, especially in the case of the aptamer, it may be difficult to determine the best position of the tag in the absence of structural information about the RNA or prior knowledge of where the proteins may bind. As an alternative to tagged RNA, antisense RNA can be used to capture the native RNA. Usually, the antisense RNA is immobilized onto a solid support and the interacting RBPs are eluted either by application of denaturing conditions or competitor oligonucleotides (Soeno et al., 2010). Antisense RNA capture enables use of the native RNA and avoids the complications of tagging RNA. However, the design of oligonucleotides to efficiently bind the single‐stranded regions of the native RNA is not simple and ideally should be informed by thermodynamic prediction of oligonucleotide and target RNA binding affinity (Walton, Stephanopoulos, Yarmush, & Roth, 2002).

Earlier RNA‐centric methods, such as RNA affinity in tandem (RAT; Hogg & Collins, 2007), captured in vitro interactions by immobilizing the bait RNA on a solid support and purifying the riboprotein complexes from cell lysates. Following washing steps to remove nonspecific interactions, RBPs are released and analyzed by MS. The advantage of this approach is that the entire ribonucleoprotein may be identified, however by re‐capitulating interactions ex vivo, it is possible to form RNA–protein interactions that do not occur in vivo. To avoid these experimental artifacts, UV crosslinking may be employed to “freeze” the in vivo interactions. Related protocols, such as chromatin isolation by RNA purification (ChIRP; Chu, Quinn, & Chang, 2012), capture hybridization analysis of RNA targets (CHART; Simon, 2013) and identification of direct RNA‐interacting proteins (iDRiP; Minajigi et al., 2015), all utilize in vivo crosslinking, pull‐down of the native RNA of interest via biotinylated antisense RNA, and protein identification by MS.

RBPs usually bind degenerate RNA motifs and multiple RNAs. To elucidate the function of an RBP, it is crucial to identify not only the RNAs it binds, but also the exact positions of interaction. Methods to study the global RNA binding sites of RBPs utilize immunoprecipitation (IP) of the native protein or an epitope tagged fusion protein. Early high‐throughput experiments combined RBP immunoprecipitation (RIP) with microarrays or sequencing (RIP‐Chip and RIP‐Seq; Cloonan et al., 2008; Penalva, Tenenbaum, & Keene, 2004) to perform genome‐wide analysis of protein‐RNA interactions. However, since RIP simply identifies the RNAs that are precipitated along with a protein, the specificity is low, leading to frequent identification of indirect and secondary interactions (Mili & Steitz, 2004; Trifillis, Day, & Kiledjian, 1999). Furthermore, standard RIP possesses poor resolution, identifying the RNA but not the binding site, however DO‐RIP‐seq now enables the identification of binding sites as well as quantification of binding site strength (Nicholson, Friedersdorf, & Keene, 2016). Specificity and resolution were both improved by the development of UV crosslinking and IP (CLIP; Darnell, 2010; König, Zarnack, Luscombe, & Ule, 2012; Licatalosi et al., 2008). Crosslinking enables more stringent wash steps to exclude indirect and secondary interactions and, by sequencing of the fragmented RNA (CLIP‐Seq), the binding site of the RBP on the RNA can also be established, with the resolution dependent on the length of the fragmented RNA. Theoretically it is possible to determine the precise site of binding as crosslinking induces point mutations and the crosslinked base causes short deletions in the cDNA due to disruption of reverse transcription (Urlaub, Hartmuth, & Lührmann, 2002; Zhang & Darnell, 2011). However, in practice, this is only possible for RNAs where the RNAseq data is of sufficiently high read depth. Two further methods have therefore been developed to fully realize single nucleotide resolution analysis. Photoactivatable ribonucleoside‐enhanced CLIP (PAR‐CLIP) extends the crosslink‐dependent mutation approach by utilizing photoactivatable nucleoside analogues (Hafner et al., 2010). These analogues can be crosslinked by UV irradiation at 365 nm and lead to a base transition during the reverse transcription at the precise point of crosslinking. Importantly, these analogues can be introduced to the cell media, where they are readily taken up by the cells and incorporated into newly synthesized transcripts, thus enabling genome‐wide single nucleotide resolution analyses. The alternative approach, individual nucleotide resolution CLIP (iCLIP), uses a modified library preparation to take advantage of the disruption of reverse transcription at the crosslink site to yield circularized cDNA from which the exact site of crosslinking can be determined (Huppertz et al., 2014; König et al., 2010). A recent modification to the iCLIP protocol, enhanced CLIP (eCLIP), simplifies the iCLIP protocol to help avoid low‐complexity libraries and experimental failure, and introduces a control sample to improve specificity (Van Nostrand et al., 2016). The improved ease by which eCLIP experiments may be performed was demonstrated by applying it to 73 diverse RBPs across 2 cell lines (Van Nostrand et al., 2016). Additionally, target RNA can be identified in the absence of protein purification and crosslinking by RNA tagging (Lapointe, Wilinski, Saunders, & Wickens, 2015). This technique utilizes a poly(U) polymerase, such as PUP‐2 from *Caenorhabditis elegans*, which is fused to an RBP of interest and covalently tags bound RNA that can be identified by high‐throughput sequencing (Lapointe et al., 2015).

There are also several approaches that allow the RNA binding sites on RBPs to be determined. One approach uses a combination of proteolytic digestion of RNA–protein conjugates isolated by RBP‐capture followed by a second round of oligo(dT) enrichment (Castello et al., 2016) and proteolysis, to release the peptide sequences adjacent to the site of cross‐linking. These “N‐link” (neighboring) peptides can then be identified by MS. As UV‐induced crosslinking forms a covalent bond between an amino acid's side chain and the nucleotide base of the RNA, a combination of RNase and proteolysis leaves peptides from RBPs where one or more amino acid remains modified by a cross‐linked base. Mass spectrometric analysis can then be used to identify the peptide sequence site, and identity of the modification. The assignment of the RNA‐crosslink site in protein digests can be challenging, as any amino acid side chains (except for Asn, Glu, and Gln) may be covalently bound to RNA upon UV‐irradiation leading to mass heterogeneity of the nucleotide remnant. Recently a promising high‐throughput computational approach has been described. The workflow, RNPxl, removes all tandem mass spectra not corresponding to cross‐linked species, and then calculates the masses of all possible peptide‐RNA conjugates followed by a database search (Kramer et al., 2014).

## 3 RNA‐BINDING DOMAINS

Currently, over 1000 RBPs with diverse cellular functions have been identified (Baltz et al., 2012; Beckmann et al., 2015; Castello et al., 2012, 2016; He et al., 2016), often recruiting larger protein complexes to RNA in response to the appropriate cellular signal (Müller‐McNicoll & Neugebauer, 2013). The binding of RBPs to RNA is mediated by RNA binding domains (RBDs), which recognize specific RNA sequences or structures. Presently it is estimated that over 40 annotated RBDs exist, encompassing both canonical and noncanonical domains (Müller‐McNicoll & Neugebauer, 2013). Examples of basic canonical RBDs found in mammalian cells include RNA recognition motif (RRM), K‐homology domain (KH domain), zinc finger binding domain (ZnF) and double‐stranded RBD (dsRBD). Although the structure of RBDs and mechanism of binding to RNA is beyond the scope of this review (extensively reviewed in (Auweter, Oberstrass, & Allain, 2006; Lunde, Moore, & Varani, 2007; Valverde, Edwards, & Regan, 2008)), it is important to note that binding is dependent on the recognition of short specific nucleotide sequences and RNA structure (Auweter et al., 2006; Lunde et al., 2007). Individually, RBDs display relatively weak affinity for a small number of nucleotides, however, RBPs employ a remarkable tactic of RBD multimerization. By utilizing multiple copies of the same RBD, RBPs enhance RNA specificity and binding affinity by increasing the number of recognized nucleotides and area of RNA binding. Furthermore, distribution of RBDs throughout an RBP allows the recognition of nucleotides separated within the RNA sequence. For example, the RRM is the most common RBD in eukaryotes and estimated to be present in 0.5–1% of genes (Cléry, Blatter, & Allain, 2008; Lunde et al., 2007). A single RRM only recognizes 2–8 nucleotides, however the presence of four RRMs, such as those found in polypyrimidine‐tract‐binding protein (PTB), enables the recognition of nucleotides at different locations within the RNA, and facilitates restructuring of the RNA itself (Sawicka, Bushell, Spriggs, & Willis, 2008). Alternatively, some RBPs combine multiple types of RBDs to enhance binding specificity. For example, CPEB proteins contain two RRMs which cooperate with a ZnF domain to promote RNA binding (Afroz et al., 2014). RBPs also combine RBDs with protein binding domains, such as in poly(A) binding protein (PABP), which contains four RRMs and a PABP binding domain (Burd, Matunis, & Dreyfuss, 1991; Deo, Bonanno, Sonenberg, & Burley, 1999). Moreover, RBDs can be partnered with kinase domains, such as those found in PKR (double‐stranded RNA‐activated protein kinase), where the binding of RNA at two dsRNA RBDs triggers structural rearrangements allowing dimerization and kinase activation (Cosentino et al., 1995). Cooperative binding of different RBPs also expands RNA sequence recognition. This is demonstrated in *Drosophila*, where Sex‐lethal (SXL) recruits UNR to the 3′ UTR of msl‐2 mRNA, enhancing the RNA binding activity of UNR and inhibiting msl‐2 translation (Hennig et al., 2014). These features enable RBPs to function in a specific manner and carry out essential cellular functions, and as such, their activities can be abrogated or dysregulated in disease. For example, mammalian UNR has been found to be overexpressed in melanoma and to specifically promote invasion and metastasis by regulating pro‐metastatic factors (Wurth et al., 2016). The important roles played by RBPs is further exemplified by the impact of a single point mutation within the KH domain of FMR1 impairing RNA binding activity and leading to the development of fragile X syndrome (Siomi, Choi, Siomi, Nussbaum, & Dreyfuss, 1994; Siomi, Siomi, Nussbaum, & Dreyfuss, 1993).

Although computational prediction of RBPs (based on sequence and structural homology of canonical RBDs) have been informative (Gerstberger, Hafner, & Tuschl, 2014; Ray et al., 2009; Zhao, Yang, & Zhou, 2011), large‐scale in vivo proteomic studies have now identified a significant number of proteins with RNA‐binding activity that lack canonical RBDs (Baltz et al., 2012; Beckmann et al., 2015; Castello et al., 2012; Castello et al., 2016; He et al., 2016). Interestingly, many of these newly identified proteins were already known to function in non‐RNA related pathways, such as metabolic enzymes (Castello et al., 2012), and has led to increased research activity to identify “moonlighting” RBPs. However, a more intriguing question concerns how proteins lacking canonical RBDs bind to RNA. Two recent studies have mapped RNA binding sites to intrinsically disordered globular regions within RBPs, termed noncanonical RBDs, using alternative techniques based on RBP‐capture (RBDmap; Castello et al., 2016); and photoactivatable nucleoside analogue incorporation (RBR‐ID; He et al., 2016). Although the nature of RBDmap may induce bias towards polyadenlyated RNA and RBR‐ID may be prone to identification of false‐positive RBPs, these radically different approaches independently confirm the presence of functional noncanonical RBDs. Despite the mechanism of RNA binding to noncanonical RBDs requiring further investigation, noncanonical RBDs have been shown to be subject to extensive post‐translational modifications (Castello et al., 2016) that may be regulated in response to cellular signaling and stress response pathways. These findings raise the possibility that noncanonical RBPs may be modified to induce structural changes that enhance RNA binding when required, and may serve to recruit other proteins to RNA and RNPs (Castello et al., 2016). Importantly, these studies create a platform to delve deeper into the role of noncanonical RBDs and will surely yield valuable insights into basic biological function. Moreover, the identification of 86 RBPs as being associated with human Mendelian disease (Castello et al., 2012), and more importantly that mutations are often found within noncanonical RBDs (Castello et al., 2016; Castello, Fischer, Hentze, & Preiss, 2013), creates an exciting opportunity to identify novel therapeutic targets (Table 1).

**Table 1:** RNA binding domains, the target mRNAs and associated disease

RBP	RBDs	RNA recognized	Associated disease
LARP1	1× RRM, 1× La motif	mRNA (TOP‐mRNA)	Tumorigenesis (Lung, ovarian cancer)
PABP	4× RRM	Poly(A) RNA	Oculopharyngeal muscular dystrophy, tumorigenesis (Breast, ovarian, CRC)
TIA‐1/TIAR	3× RRM	mRNA	Tumorigenesis (tumor suppressors)
PKR	2× dsRBD	dsRNA	Neurodegenerative disease (Huntington, Alzheimer's disease)
PTB	4× RRM	pre‐mRNA, mRNA	Tumorigenesis (Breast, ovarian, CRC)
FMR1	2× KH‐domain	mRNA	Fragile X syndrome
Nucleolin	4× RRM	pre‐rRNA, rRNA, mRNA	Tumorigenesis
HuR	3× RRM	mRNA	Hu syndrome

### 3.1 RNA motifs and their role in translation regulation

**Figure 1:**
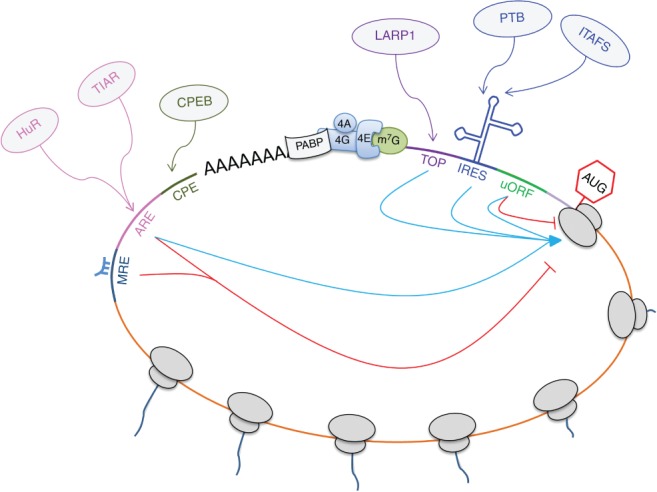
RNA motifs and interactions with *trans*‐acting factors. Schematic representation of translational regulation mediated by the binding of *trans*‐acting factors (in grey) to *cis*‐acting elements along the pseudo‐circularized messenger RNA. Terminal oligopyrimidine motifs (TOPs), internal ribosome entry sites (IRESs) and uORFs in the 5′ UTR; and miRNA‐responsive (MREs), AU‐rich (AREs) and cytoplasmic polyadenylation (CPEs) elements in the 3′ UTR, are known to either stimulate or inhibit cellular protein synthesis. AUG indicates the start of the ORF

Whereas global protein synthesis control is mainly exerted through changes in the phosphorylation status of eIFs and eEFs, the recruitment of *trans*‐acting factors to *cis*‐acting elements in the 5′ and 3′ UTRs has a major impact on multiple aspects of mRNA metabolism, including polyadenylation, nuclear export, transcript localization, stability, and consequently protein synthesis (Martin & Ephrussi, 2010). Some of most common or frequently used RNA motifs are discussed below, including TOP motifs, upstream open reading frames approximately (uORFs), internal ribosome entry segments (IRES), cytoplasmic polyadenylation elements (CPEs), AU rich elements, and microRNA target sites (Figure 1).

### 3.2 Terminal oligopyrimidine motif

Terminal oligopyrimidine (TOP)‐containing mRNAs contain a motif following their 7‐methylguanosine triphosphate (m7 GTP) cap, which consists of an invariable C‐residue, followed by a 4–15 base pyrimidine tract containing a similar proportion of Cs and Us (Meyuhas & Kahan, 1849). A CG‐rich region is often found immediately downstream of the 5′ TOP motif and it is thought that in some cell types both the TOP motif and the CG‐rich region are required for full translational control (Avni, Biberman, & Meyuhas, 1997; Avni, Shama, Loreni, & Meyuhas, 1994). Studies to identify the number of TOP‐containing mRNAs have suggested that there are 93 such mRNAs including 79 out of 80 ribosomal proteins, all five translation elongation factors (*EEF1A1*, *EEF1B2*, *EEF1D*, *EEF1G*, *and EEF2*), translation initiation factors (eIF3e, eIF3f, eIF4B, and eIF3h), poly(A) binding protein (PABPC1), nucleophosmin (NPM1), receptor of activated protein C kinase 1 (Rack1/GNB2L1), translationally‐controlled tumor protein 1 (TPT1), polypyrimidine tract‐binding protein 1 (PTPB1/HNRNP1), nucleosome assembly protein 1‐like 1 (NAP1L1) and vimentin (VIM; Horvilleur et al., 2013; Iadevaia, Caldarola, Tino, Amaldi, & Loreni, 2008; Meyuhas & Kahan, 1849; Yoshihama et al., 2002). mRNAs that contain TOP‐motifs are translated in response to cell growth stimuli such growth factors and insulin (Patursky‐Polischuk et al., 2014), and in contrast, their translation is rapidly repressed following amino acid and serum starvation (Granneman & Tollervey, 2007; Hornstein & Meyuhas, 2001; Ørom, Nielsen, & Lund, 2008; Pardee, 1989). Despite more than two decades of research, the precise complement of *trans*‐acting factors that control the translation of TOP‐containing mRNAs are still unidentified (Meyuhas & Kahan, 1849), although a number of studies have shown that LARP1 (see below) has a major role in their regulation (Shama & Meyuhas, 1996).

### 3.3 Upstream open reading frames

**Figure 2:**
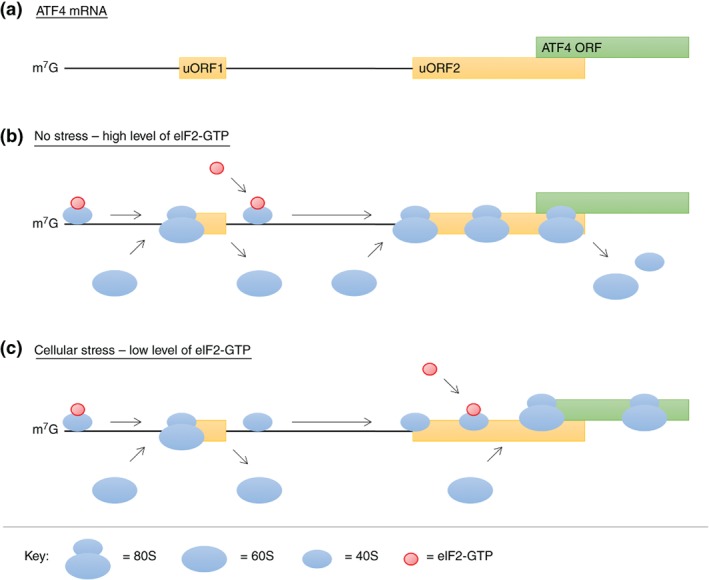
uORFs and cellular stress. ATF4 is a transcription factor regulated at the level of transcription and translation in response to stress (Dey et al., 2010), and plays an important role in the integrated stress response (ISR) by enhancing the expression of stress response transcripts (Harding et al., 2000; Vattem & Wek, 2004; Wek, Jiang, & Anthony, 2006). ATF4 mRNA translation is mediated by a delayed re‐initiation mechanism at two uORFs (Vattem & Wek, 2004). (a) ATF4 uORF1 is very short (encoding three amino acids) and functions in a positive manner, enhancing re‐initiation at downstream uORF2 (encoding 59 amino acids) which overlaps with the ATF4 ORF. (b) Under normal conditions, when eIF2‐GTP (and thus TC) availability is high, ribosomes translate uORF1 and the 60S ribosome dissociates. Importantly, the 40S ribosome remains associated and continues to scan the message, reinitiating at the inhibitory uORF2 and bypassing the ATF4 CDS (inhibiting ATF4 translation). (c) In response to cellular stress, when eIF2 is phosphorylated and eIF2‐GTP (and thus TC) availability is low, the scanning 40S ribosome bypasses uORF2 and instead re‐initiates at the start of the ATF4 ORF, increasing ATF4 translation. Therefore, delayed re‐initiation provides a mechanism for cells to selectively enhance the translation of mRNAs, particularly those encoding proteins required for adaptation and recovery from stress, during global protein synthesis inhibition

Translation of cellular mRNAs generally occurs via cap‐dependent scanning (Hinnebusch, Ivanov, & Sonenberg, 2016) and initiates at AUG start codons located within the Kozak sequence (5′‐(A/G)CCAUGG‐3′) (Kozak, 1987). AUG start codons within the 5′ UTR of mRNAs give rise to uORFs (Hinnebusch et al., 2016), which are small, conserved, repressive elements that inhibit the translation of mRNA open reading frame (ORF; Chew, Pauli, & Schier, 2016; Johnstone, Bazzini, Giraldez, & Upstream, 2016), and have been shown to be present in almost 50% of protein coding transcripts (Wethmar, Barbosa‐Silva, Andrade‐Navarro, & Leutz, 2014). The elements function during conditions of cell stress, when global protein synthesis is downregulated through the phosphorylation of eIF2, which decreases the availability of eIF2‐GTP and thus TC (Figure 2). In mammalian cells, phosphorylation of eIF2 is mediated by four eIF2 kinases (eIF2Ks): PERK, GCN2, PKR, and HRI, which are activated in response to a range of stresses such as ER stress, amino acid starvation, DNA damage, dsRNA and heme deficiency (Donnelly, Gorman, Gupta, & Samali, 2013). Recent advances in ribosome profiling techniques have enabled the transcriptome wide study of uORFs in unprecedented scale, identifying the location and regulatory function of uORFs within mRNA. Importantly, these studies have all strongly supported the notion that uORFs are involved in active translation (Chew et al., 2016; Ingolia, Lareau, & Weissman, 2011; Johnstone et al., 2016), functioning as important regulators of mRNA translation in response to cellular stress. Moreover, further technical advances and improved single codon resolution has the potential to yield valuable insight into the function and regulation of uORFs in vivo. However, given that 50% of mRNAs contain uORFs and the selective upregulation of translation of uORF‐containing mRNAs is dependent on the cell stress imposed, it is likely that their specific translation is mediated by RBPs, although these have yet to be identified.

### 3.4 Internal ribosome entry segments

IRES are elements present in the 5′‐UTR of viral mRNAs permitting cap‐independent initiation of translation (Jang et al., 1988; Pelletier, Kaplan, Racaniello, & Sonenberg, 1988) and their function and mechanism of action have been extensively reviewed (Lee, Chen, & Shih, 2017). IRESs have also been identified in the 5′ UTRs of specific cellular mRNAs and the data suggest that they are present in approximately 10% of all mRNAs (Mitchell et al., 2005; Weingarten‐Gabbay et al., 2016). Cellular IRESs allow recruitment of the ribosome at a site downstream from the 5′ end of the mRNA (Holcik, Lefebvre, Yeh, Chow, & Korneluk, 1999; Johannes & Sarnow, 1998; Stoneley, Paulin, Le Quesne, Chappell, & Willis, 1998), but are very distinct from viral IRESs in that many mRNAs that have the capacity to initiate translation via an IRES are also translated by cap‐dependent scanning. The data suggest that IRESs are used under conditions of stress. For example, during early apoptosis, cap‐dependent translation is inhibited by de‐repression of eIF4E–BP and phosphorylation of eIF2, which prevents pre‐initiation complex assembly at the mRNA 5′‐cap by reducing the amount of available eIF4E and TC (Morley, Coldwell, & Clemens, 2005). Under these conditions, specific IRESs function to allow execution of this process (Bushell et al., 2006). Cellular IRESs require *trans‐*acting factors (ITAFs) for their activity (King, Cobbold, & Willis, 2010; Mitchell, Spriggs, Coldwell, Jackson, & Willis, 2003) and IRES‐mediated mRNA translation differs among cell lines, which has been shown to correlate with differential expression of ITAFs (Stoneley et al., 2000).

### 3.5 Poly (A) tails

Translation initiation is stimulated by the presence of a poly(A) tail and it has been shown that the length of the poly(A) tail directly affects the rates of mRNA translation; mRNAs with short poly(A) tails (<50 A) are generally translationally repressed. Regulation of poly(A) tail length therefore provides both global and specific control of translation. This process is critical during oogenesis, where subsets of mRNAs with short poly(A) tails are stored in a translationally repressed state and then translationally activated by polyadenylation at specific stages of oocyte or early embryonic development (de Moor, Meijer, & Lissenden, 2005). Poly(A) tails are bound by PABPs which control translation in a number of ways including recruitment of release factors during the termination process (Ivanov et al., 2016; see below).

### 3.6 Cytoplasmic polyadenylation elements

CPEs are pyrimidine‐rich hexanucleotides with a canonical 5′‐UUUUA1‐2U‐3′ sequence present in the 3′ UTRs 20–30% of vertebrate genes (Piqué, López, Foissac, Guigó, & Méndez, 2008). CPEs are bound by cytoplasmic polyadenylation element binding proteins (CPEBs) which control the translation of specific mRNAs through the activation and repression of polyadenylation (Ivshina, Lasko, & Richter, 2014).

### 3.7 AU‐rich elements

Among the most studied 3′ UTR elements are the AU‐rich elements (AREs). These actively participate the post‐transcriptional control, through the cooperation or competition between *trans‐*acting factors binding the AUUUA pentamers. Numerous genes including those encoding cyclins, transcription factors, tumor suppressors and oncogenes are regulated by AREs (Eberhardt, Doller, Akool, & Pfeilschifter, 2007). Therefore, any mutation in both *cis*‐ and *trans‐*acting elements has a negative impact on cell growth and development. A recent comprehensive reanalysis of CLIP data in HEK293 cells reported a significant number regulatory motifs within the 3′UTRs are AREs (Plass, Rasmussen, & Krogh, 2017). Interestingly, the 3′UTRs of transcripts encoding transcriptional and post‐transcriptional regulatory factors are among the most enriched for AREs, in agreement with previous studies where the interactions of RBPs with such 3′ UTR elements were shown to create auto‐regulatory networks (Stoiber et al., 2015). The ARE‐binding proteins (ARE‐BP) are divided into those responsible for mRNA stabilization and translation enhancement (e.g., ELAV family, NF90), and those increasing RNA decay and translation repression (e.g., TTP, TIAR, TIA1, AUF1, BRF1, KSRP). The function of this protein family in the regulation of translation has been described extensively elsewhere (Garneau, Wilusz, & Wilusz, 2007) and will not be discussed further in this review.

### 3.8 MicroRNA target sites

MicroRNAs (miRNAs) are conserved 21–23 nt ncRNA molecules that have regulatory roles in development, differentiation, proliferation, and metabolism (Bartel, 2009). Their biological function is exerted by forming competent miRNA‐induced silencing complexes (miRISCs) and partially or totally base‐pairing with miRNA‐responsive elements (MRE) in the 3′ UTR of mRNA targets. In so doing, miRNAs repress mRNA translation and accelerate their degradation in concert with deadenylase, decapping, and 5′‐3′ decay complexes. Therefore, microRNAs can be considered key *trans*‐acting elements in the post‐transcriptional control. However, the subject of microRNAs and their interaction with mRNA and RNA‐binding proteins (RBPs) has been reviewed extensively elsewhere and is therefore not included here (Chang & Hla, 2017; Ciafrè & Galardi, 2013; Jiang & Coller, 2016; Maroney, Yu, & Nilsen, 2006).

## 4 RNA BINDING PROTEINS AND THEIR ROLE IN TRANSLATION

As described earlier in this review, from transcription and processing to export and translation, mRNAs are bound by ever changing complements of proteins. Subsequently, RBPs coordinate many aspects of the mRNA life cycle such as stability, localization and translation (Lunde et al., 2007; Müller‐McNicoll & Neugebauer, 2013), and provide an additional layer of post‐transcriptional regulation in response to cellular stress (Harvey, Dezi, Pizzinga, & Willis, 2017). In this section we discuss four representative mammalian RBPs in detail, namely LARP1, PTB/hnRNPI, PABP, and CPEB, which are known to have both positive and negative effects on the translation of a large number mRNAs by interacting with specific RNA motifs.

### 4.1 La‐related protein 1

**Figure 3:**
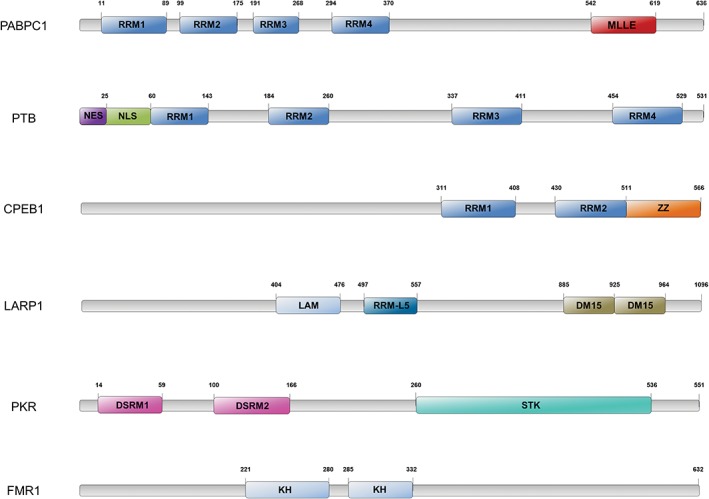
Comparison of selected RNA‐binding proteins and their domains. Comparison of selected RNA‐binding proteins and their domains. PABPC1 (polyadenylate‐binding protein cytoplasmic 1); PTB (polypyrimidine tract‐binding protein 1); CPEB1 (cytoplasmic polyadenylation element‐binding protein 1); LARP1 (La‐related protein 1); PKR (double‐stranded RNA‐activated protein kinase); FMR1 (Fragile X mental retardation protein 1). Domains: RRM (RNA recognition motif); MLLE (Met‐Leu‐Leu‐Glu motif); NES (nuclear export sequence); NLS (nuclear localization sequence); ZZ (ZZ‐type zinc finger domain); LAM (La motif); RRM‐L5 (RRM‐like motif 5); DSRM (double‐stranded RNA‐binding motif); STK (Ser‐Thr kinase domain); KH (K homology RNA‐binding domain)

La‐related protein 1 (LARP1) is an evolutionarily conserved RNA binding protein with predominantly cytoplasmic expression. It contains the well–conserved La motif, a 90‐amino acid domain, which is followed by an RNA recognition motif (RRM‐L5) and a highly conserved C‐terminal region, comprised of triplicate amino acid repeats. This region was originally termed the DM15 domain (Bousquet‐Antonelli & Deragon, 2009), but is now also referred to as the LARP1 motif (Stavraka & Blagden, 2015) (Figure 3). LARP1 contains binding sites for PABP (Blagden et al., 2009) and regulatory‐associated protein of mTOR (RAPTOR) and through these protein/protein interactions, in addition to direct RNA binding, LARP1 controls the translation of subsets of mRNAs. Thus a number of studies have shown that LARP1 plays an important role in the regulation of mRNAs that contain 5′ TOP motifs.

Mechanistic target of rapamycin (mTOR) controls the translation of 5′ TOP‐containing mRNAs (Jefferies et al., 1997) and this is in part due to the association of LARP1 with RAPTOR and PABP, and the regulation of LARP1 activity by signaling through mTOR (Aoki et al., 2013; Burrows et al., 2010; Fonseca et al., 2015). Interestingly, both positive and negative regulatory functions have been attributed to LARP1 in terms of its effects on TOP‐mRNA translation (Burrows et al., 2010;Fonseca et al., 2015 ; Tcherkezian et al., 2014). It has been shown that LARP1 associates directly with the 5′ TOP motif via the DM15 region (Lahr et al., 2015) and recent crystallographic data has demonstrated that this region of LARP1, by specifically binding to the m7GTP cap and the first cytidine of TOP mRNAs, prevents eIF4E from interacting. These interactions block the assembly of eIF4F complex on TOP‐containing mRNAs (Lahr et al., 2017). The in vitro data also showed that LARP1 had a higher affinity for TOP‐containing mRNAs than eIF4E, which is entirely consistent with a role as a repressor of translation (Fonseca et al., 2015). However, it appears that LARP1 function may be dependent on the cell context and that it interacts with additional RNA motifs. For example, in cancer cells LARP1 has positive effects on total protein synthesis (Burrows et al., 2010) and binds to a range of targets including those that encode proto‐oncogenes (Hopkins et al., 2016; Mura et al., 2015) in addition to TOP‐containing mRNAs (Fonseca et al., 2015; Tcherkezian et al., 2014). Thus the data suggest that while LARP1 is able to repress TOP‐containing mRNA translation by binding to the 5′ TOP motif, it is also capable of activating the translation of other targets (Lahr et al., 2017). In agreement with this notion, a recent study using PAR‐CLIP in HEK‐293 cells showed that LARP1 interacts directly with pyrimidine rich sequences which overlap with the TOP‐motif, but also interact with the 3′ UTRs of mRNAs that encode ribosomal proteins. These alternative modes of binding were controlled by phosphorylation through mTORC1 and Akt/S6K1. Phosphorylation of LARP1 caused its dissociation from 5′ polypyrimidine rich sequences, but stimulated binding to the 3′ UTRs of mRNAs encoding ribosomal proteins (Hong et al., 2017). These data suggest that LARP1 acts as a phosphorylation sensitive molecular switch activating or repressing cell proliferation in response to environmental cues (Hong et al., 2017).

Given that LARP1 is able to control translation of subsets of mRNAs that are central to cell proliferation, it is unsurprising that aberrant expression is associated with tumorigenesis (Mura et al., 2015). These data suggest that elevated levels of LARP1 are associated with a variety of cancers (Burrows et al., 2010; Xie et al., 2013); for example in cervical cancer, LARP1 protein expression correlates with increasing disease progression whereby stepwise elevations in LARP1 expression are observed from the pre‐invasive stages (CIN1–3) into invasive disease (Burrows et al., 2010; Hopkins et al., 2016). It is unclear what drives LARP1 expression in this context, although cancer cells are often exposed to aberrant growth factor signaling. In addition, there are two isoforms of LARP1, measuring 1019 and 1096 amino acids respectively, that differ not only in length, but also in their N‐terminal sequences. Whether one isoform contributes more to LARP1 function in malignant cells than another has yet to be determined.

### 4.2 PTB/hnRNP 1

PTB (poly pyrimidine tract binding protein—also known as hnRNP I) is a ubiquitously expressed RBP that specifically binds polypyrimidine tracts (CU‐rich elements) within pre‐mRNA and mature mRNA (Oberstrass, Hargous, Henning, & Wenter, 2005). PTB binds RNA via four RRMs (Figure 3) (Oh et al., 1998), although it has been suggested that RRM‐3 and RRM‐4 (within the C‐terminal domain) exhibit the strongest RNA binding activity (Oh et al., 1998) (Figure 3). Moreover, mutations specifically impairing the RNA binding activity of RRM‐4 reduce PTB‐dependent function, suggesting that RRM‐4 harbors some of the most important RNA interacting sites (Mickleburgh et al., 2014). PTB regulates many aspects of RNA biology including splicing, localization and stability and mRNA translation. PTB is a central regulator of splicing, however only 33% of genome wide PTB binding sites are linked to splicing (Xue et al., 2009). Nuclear export‐and localization‐sequences within the N‐terminal domain (Figure 3) allows PTB to shuttle to the cytoplasm where it binds extensively to viral‐and cellular‐mRNA (King et al., 2014; Sawicka et al., 2008), as well as mitochondrial tRNA^Thr^ (mt tRNA^Thr^; Marnef, Jády, & Kiss, 2015).

PTB is required for translation mediated by viral and cellular IRESs (Kafasla, Lin, Curry, & Jackson, 2011; Mitchell et al., 2005) and binding of each RRM to different regions of a single RNA enables extensive restructuring of the 5′ UTR is essential for this process (Kafasla et al., 2011; Sawicka et al., 2008). Additionally, PTB forms complexes with proteins including PTB‐associated splicing factor (PSF), YBX1 and NONO, recruiting them to RNA (King et al., 2014). PSF has recently been identified to contain an IRES element within the 5′ UTR of its mRNA, and intriguingly, PTB enhances IRES‐mediated PSF translation during viral infection (Dave, George, Sharma, & Das, 2017), suggesting PTB can regulate the expression of its binding partners.

The combinatorial binding of RBPs can regulate the fate of mRNA in response to stress or stimulation. For example, during TNF‐related apoptosis inducing ligand (TRAIL)‐mediated apoptosis, PTB accumulates within the cytoplasm and the composition of PTB‐containing protein complexes are remodeled to regulate the rate of apoptosis, and enhance the translation of PTB‐dependent IRESs (King et al., 2014). PTB has also been show to stimulate cap‐dependent translation and binding of PTB and TIAR to insulin mRNA in response to glucose stimulation is associated with enhanced insulin synthesis in human pancreatic cells (Fred, Mehrabi, Adams, & Welsh, 2016). Depletion of PTB reduces cellular levels of insulin mRNA, whereas depletion of TIAR enhances insulin mRNA levels (Fred et al., 2016), indicating the stability and translation of mRNA can be regulated by differential binding of subsets of RBPs.

In common with other RBPs, PTB has been directly implicated in disease progression (Castello, Fischer, et al., 2013) and has been found to be overexpressed in ovarian cancer, enhancing tumor growth and invasiveness (He et al., 2007). Moreover, PTB expression is enhanced in breast cancer cell lines, contributing to cell growth and malignant behavior, implicating PTB in breast cancer tumorigenesis (He et al., 2014).

### 4.3 Poly(A)‐binding proteins

Poly(A)‐binding proteins (PABPs) belong to a highly conserved family of eukaryotic RBPs involved in various stages of post‐transcriptional regulation of gene expression, including pre‐mRNA 3′‐end processing, translation initiation, termination, mRNA stability and turnover, and mRNA‐specific degradation mechanisms (Brook & Gray, 2012; Chan, Choi, & Shi, 2010; Ivanov et al., 2016; Smith, Blee, & Gray, 2014; von der Haar, Ball, & McCarthy, 2000). PABP is imported into the nucleus where it binds to a poly(A) mRNA via its N‐terminal RRM cluster (Figure 3) and is exported with the mRNA ribonucleoprotein particle (Hosoda, Lejeune, & Maquat, 2006; Kühn & Pieler, 1996). PABP was first identified as a protein that protected mRNA poly(A) tails from deadenylation, which precedes de‐capping and 5′‐3′ degradation (Bernstein, Peltz, & Ross, 1989). However, PABP has a number of roles in the regulation of translation, the best‐characterized of which is its impact on cap‐dependent translation initiation. PABP interacts with the eIF4F cap‐binding complex via the eIF4G subunit, which has been shown to increase eIF4E cap‐binding affinity, and eIF4A helicase activity, required for resolving structures in the 5′‐UTR of mRNAs during 43S scanning (Bi & Goss, 2000; Svitkin et al., 2001; Wei, Balasta, Ren, & Goss, 1998). It is believed that PABP, by virtue of its interaction with eIF4G and poly(A), stabilizes bound mRNAs into a closed loop conformation such that it enhances both pre‐initiation complex assembly and post‐termination ribosome recycling (Rajkowitsch, Vilela, Berthelot, Ramirez, & McCarthy, 2004; Tarun & Sachs, 1996).

PABP can be regulated by PABP‐interacting proteins 1 and 2 (Paip1 and 2). Paip1 binds PABP and probably enhances its affinity for eIF4G, while Paip2 is a competitive inhibitor of PABP‐eIF4G interaction (Karim et al., 2006; Martineau et al., 2008) and can inhibit the interaction between PABP and the poly(A) tail (Khaleghpour et al., 2001). The importance of Paip2 regulation of PABP is evident in spermiogenesis, during which transcription is silenced and gene expression is mostly governed by translational control (Braun, 1998). Failure to express Paip2 in mice causes infertility, decreased sperm count, and abnormal spermatid ultrastructure, which is correlated with changes in the expression profile of PABP (Yanagiya, Delbes, Svitkin, Robaire, & Sonenberg, 2010). Mechanistically it has been suggested that these defects are due to excessive levels of PABP leading to nonproductive eIF4G binding and/or competitive binding to mRNA 5′‐UTRs, while in normally developing spermatozoa PABP activity is modified by Paip2 (Eliseeva, Lyabin, & Ovchinnikov, 2013; Yanagiya et al., 2010). Complementary proteomic analysis of Paip2 knockout spermatids suggests that the latter mechanism exerts translational control on a subset of mRNAs, and may be due to specific *cis*‐acting elements (Delbes, Yanagiya, Sonenberg, & Robaire, 2012). This notion is plausible, given that multiple proteins have been reported to regulate the translation of mRNAs containing specific UTR elements via PABP (Collier, Gorgoni, Loveridge, Cooke, & Gray, 2005; Kawahara et al., 2008; Reynolds et al., 2005).

PABP has an important role in regulating mRNA translation following viral infection, although different classes of viruses vary considerably in their dependence on PABP. Thus certain viral proteases, such as EMCV 3C proteinase, cleave PABP and inhibit host cell protein synthesis to allow selective synthesis of viral proteins (Joachims, Van Breugel, & Lloyd, 1999; Kerekatte et al., 1999; Kuyumcu‐Martinez, Van Eden, Younan, & Lloyd, 2004). Conversely, Dengue virus 3′ UTR RNA is bound by PABP in a Paip2‐sensitive manner, which may increase its translational efficiency (Polacek, Friebe, & Harris, 2009). In contrast, Rotavirus nonstructural protein 3, which competes with PABP for eIF4G, enhances the translation of both viral and host mRNA, probably via eIF4G‐4E stabilization (Gratia et al., 2015). Further to this, PABP appears to have an emerging role in mechanisms of noncanonical translation initiation (Smith et al., 2017).

PABP has also been shown to have a role in translation termination. PABP interacts with eRF3a, which is attributed to its role in mRNP closed loop formation and nonsense‐mediated mRNA decay (Brook & Gray, 2012; Silva, Ribeiro, Inacio, Liebhaber, & Romao, 2008). Recent work has shown that PABP enhances termination complex assembly and peptide release, suggesting that its interaction with eRF3a contributes to the fidelity of stop codon recognition (Ivanov et al., 2016). Mechanistic detail of this novel function of PABP has yet to emerge, but it is speculated that PABP recruits and orients eRF3a to the terminating ribosome (Ivanov et al., 2016). These data suggest that PABP is able to enhance stop codon recognition in *trans* on a reporter mRNA lacking poly(A), although it is unclear how this occurs (Ivanov et al., 2016). These findings have particular relevance to human disease, as it has been shown that allelic variants of eRF3a are associated with increased risk of certain cancers (Brito et al., 2005; Malta‐Vacas et al., 2009; Miri, Hemati, Safari, & Tavassoli, 2011). Furthermore, recombinant eRF3a containing such alleles has been shown to bind differentially to PABP, with one particular allelic variant exhibiting an eight‐fold lower binding affinity (Jerbi, Jolles, Bouceba, & Jean‐Jean, 2016), however, a relevant pathogenic mechanism has not yet been described.

### 4.4 Cytoplasmic polyadenylation element binding proteins

CPEBs are a highly conserved family of proteins that can be clustered in two sub‐families: CPEB1 and CPEB2–4 (Wang & Cooper, 2010). All CPEB members have a common C‐terminal region that contains RRMs (Figure 3). These motifs consist of two RRMs in tandem, followed by a zinc‐binding domain with a cross‐braced zinc binding topology (ZZ domain; Afroz et al., 2014). Therefore all CPEBs recognize the same CPE containing RNAs, although with different affinities (Igea & Méndez, 2010; Novoa, Gallego, Ferreira, & Mendez, 2010; Ortiz‐Zapater et al., 2011; Pavlopoulos et al., 2011). Notwithstanding, the N‐terminal domain of CPEBs is highly variable (Wang & Cooper, 2010) and contains several regulatory domains susceptible to phosphorylation (Mendez, Hake, et al., 2000; Mendez, Barnard, & Richter, 2002), monoubiquitination (Pavlopoulos et al., 2011) or sumoylation (Drisaldi et al., 2015). The combination of these post‐translational modifications, as well as the presence of other *cis*‐acting elements, determines the activity of the different CPEBs (Belloc & Méndez, 2008; Mendez, Hake, et al., 2000; Piqué et al., 2008). Once bound to RNA, and dependent on activation state, CPEBs can recruit different protein complexes that stimulate or inhibit the elongation of the polyA tail of CPE‐containing mRNAs (Fernández‐Miranda & Méndez, 2012; Ivshina et al., 2014). Thereby, these proteins can act as post‐transcriptional switches regulating gene expression through poly(A) tail modification.

CPEB‐mediated regulation is particularly important when gene transcription is silenced, or when the transcription, translation and trafficking of a protein is too slow to respond to a given stimulus. Thus, CPEBs function is key, but not restricted, to embryonic development (O'Connell, Cavallo, & Firnberg, 2014; Ota, Kotani, & Yamashita, 2011; Racki & Richter, 2006). In *Xenopus* quiescent oocytes, CPEB1 acts as a translational repressor of maternal RNAs, through the recruitment of PARN ribonuclease (Kim & Richter, 2008). Upon progesterone stimulation of oocytes, CPEB1 is phosphorylated (by AurKA) which triggers a polyadenylation wave that is necessary for cdk1 expression, and entrance into M1 phase of the cell cycle (Mendez, Murthy, Ryan, Manley, & Richter, 2000; Piqué et al., 2008). CPEB1 is then ubiquitinated and degraded (Piqué et al., 2008; Setoyama, Yamashita, & Sagata, 2007). CPEB4, which contains a CPE in its own 3′ UTR, is translated upon CPEB1 activation and assumes control of polyadenylation in the second meiotic division. CPEB4 activation is mediated by cdk1 and ERK2, which phosphorylate 12 residues surrounding two intrinsically disordered regions (IDRs) of the protein (Guillén‐Boixet, Buzon, Salvatella, & Méndez, 2016).

CPEB is also required to regulate translation in highly polarized cells such as neurons. Interestingly, it has been shown that CPEBs are necessary for long‐term potentiation (LTP) memory processes in mice (Alarcon, 2004), *Drosophila* (Khan et al., 2015; Majumdar et al., 2012) and *Aplysia* (Heinrich & Lindquist, 2011; Miniaci et al., 2008; Raveendra et al., 2013). In nonstimulated conditions, CPEBs function as translational inhibitors by binding and repressing mRNAs in the dendrites. Upon serotonin stimulation, CPEB expression is enhanced and, once a given threshold is reached, aggregates in a physiological prion‐like state (Chen, Zheng, & Wolynes, 2016; Miniaci et al., 2008; Raveendra et al., 2013; Si, Lindquist, & Kandel, 2003). This change in conformation switches CPEBs from translational inhibitors to translational activators, allowing the translation of previously stored/repressed mRNAs. Furthermore, CPEB activation can be maintained, which aids the dendritic‐specific molecular changes which sustain LTP processes.

Given the wide range of cellular processes that require regulation through CPEBs, it is not surprising that their aberrant expression is associated with a range of pathologies, including cancers. Thus, CPEB dysregulation has been described in glioblastoma, colorectal cancer and pancreatic cancer (Chang et al., 2014; Ortiz‐Zapater et al., 2011; Villanueva et al., 2017). It can be challenging to attribute a specific role for CPEBs in disease since they are involved in complex regulatory loops, however these data show that CPEB4 has a direct role in tumor vascularization and metastasis (Ortiz‐Zapater et al., 2011), while post‐transcriptional regulation mediated by CPEBs has been shown to directly regulate tumor‐specific transgene expression (Villanueva et al., 2017). Due to these direct effects on oncogene expression it has been proposed that targeting CPEBs could provide novel therapeutic targets for treatment of cancers (Villanueva et al., 2017).

## 5 RIBOSOMAL PROTEINS FUNCTION AS *TRANS*‐ACTING REGULATORY PROTEINS

The eukaryotic 80S ribosome is a large molecular machine consisting of two unequal subunits, the small 40S and the large 60S. Each subunit is composed of ribosomal RNA (rRNA)—the 18S for the small subunit and the 25/28S, 5.8S and 5S for the large subunit—and ribosomal proteins (r‐proteins or RPs), 33 in the 40S (RPS) and 47 in the 60S (RPL). Recent advancements in structural biology, particularly cryo‐electron microscopy (cryo‐EM), have provided unprecedented detail in the understanding of the structure of the eukaryotic ribosome (Anger et al., 2013), as well as of the mechanisms of its assembly (Kressler, Hurt, & Baßler, 2017). Remarkably, an increasing body of evidence indicates that ribosomes also act as regulators of gene expression by filtering the translation of certain subpools of mRNAs, largely depending on the presence of specific ribosomal proteins. Although such biological evidence is recent, “the ribosome filter hypothesis” was originally proposed in 2002 (Mauro & Edelman, 2002), and the heterogeneity of ribosomes was reported a decade earlier (Ramagopal, 1992).

Specialized roles for ribosomal subunits primarily emerged in the context of translation of viral mRNAs containing IRESs (Landry, Hertz, & Thompson, 2009; Lee, Burdeinick‐Kerr, & Whelan, 2013; Majzoub et al., 2014). For example, 40S proteins RPS25 and RACK1 mediate cap‐independent translation initiation at the IRES of hepatitis C transcripts (Landry et al., 2009; Majzoub et al., 2014), whereas RPL40 is required for cap‐dependent translation in vesicular stomatitis virus (Lee et al., 2013). Since these proteins do not appear to be essential for general mRNA translation, they could prove to be valuable targets in anti‐viral therapies.

Other examples of translational regulation that are dependent on ribosomal heterogeneity have important implications in development. In particular, RPL38 promotes formation of the 80S on a subset of Homeobox (Hox) mRNAs in mouse embryos, with mutations in the Rpl38 gene resulting in tissue‐specific patterning defects (Kondrashov et al., 2011). Unsurprisingly, RPL38‐dependent translation of Hox mRNAs was found to rely on the presence of IRES‐like sequences within their 5′ UTRs, working in conjunction with an inhibitory element that prevents transcripts from undergoing cap‐dependent translation (Xue et al., 2015). In addition, different expression patterns were observed across tissues of the mouse embryo for most ribosomal proteins, including several high‐homology paralogs, further suggesting that ribosome composition changes in a tissue‐specific manner (Kondrashov et al., 2011). However, recent evidence shows ribosome heterogeneity exists even within the same cell type. Thus it was shown by absolute quantification of RP stoichiometry that RPS7, RPS25, RPS26, and RPL10A are not present in every ribosome in the relatively homogeneous mESCs (Shi et al., 2017). Moreover, RPs were endogenously tagged to reveal that specific ribosomes preferentially translate certain functional subpools of mRNAs (Shi et al., 2017). In particular, RPL10A‐containing ribosomes were found to preferentially translate transcripts encoding proteins involved in several cell metabolic pathways, while the presence of RPS25 seems to be associated with preferential translation of cell cycle mRNAs and, remarkably, all the mRNAs from the vitamin B12 synthesis pathway. It is particularly interesting that when transcripts from a certain process are preferentially translated by ribosomes containing a specific ribosomal protein, mRNAs from opposing processes would often appear to evade translation by the same ribosomes. For example, the RPL10A translatome shows an enrichment of mRNAs involved in cell growth or cancer metastasis and a significant depletion of transcripts important for the stress response (Shi et al., 2017). Such a strategy may allow eukaryotic cells to respond quickly and efficiently to different cues, by switching ribosome preferences rather than micromanaging single mRNAs.

In addition to ribosomal core protein heterogeneity, a complex network of ribosome‐associated proteins (RAPs) can also modulate gene expression. In another recent study aimed at investigating the ribo‐interactome in mESCs, affinity purification targeted at two core ribosomal proteins lead to the identification of several unexpected RAPs, including proteins involved in the cell cycle and enzymes from the glycolytic pathway (Simsek et al., 2017). Among these, PKM2 was found to be associated with ER ribosomes, where it might function as a translational activator. This observation opens the interesting hypothesis that different combinations of RAPs might regulate the translation of spatially restricted subpools of mRNAs at specific subcellular sites. Moreover, the association of the translation machinery with glycolytic enzymes hints at a possible strategy by which glucose homeostasis and translation rates are coordinated in eukaryotic cells.

Enzymes involved in post‐translational modification (PTM) of proteins were also identified within the ribo‐interactome where as well as modifying nascent proteins, they modify the ribosome itself in order to modulate its activity. Indeed, it has been proposed that PTMs such as phosphorylation or ubiquitylation of ribosomal proteins might represent an additional layer of translation regulation (Simsek & Barna, 2017). Interestingly, among the PTM enzymes identified was UFL1, the only known enzyme to catalyze the metazoan‐specific PTM ufmylation (Zhang et al., 2015). Certain ribosomal proteins, namely uS3, uS10, and uL1, as well as initiation factor eIF6, were indeed shown to undergo ufmylation, possibly as a strategy to coordinate subunit joining (Simsek et al., 2017).

These recent reports pave the way to the investigation of a whole new layer of gene expression regulation, with potentially extraordinary implications in the research for treatment of ribosomopathies and in all areas of cell biology.

## 6 CONCLUSIONS

Realization of the importance of the regulatory role of noncanonical *trans*‐acting proteins, and the elements within mRNA to which they bind, is advancing knowledge in the mechanisms that control translation. While there are some very well studied examples of *trans*‐acting proteins, including several ribosomal proteins, many novel RBPs have emerged as new regulatory candidates. These novel RBPs have been identified by methodologies that not only enable their identification as mRNA interactors, but also provide insight into noncanonical RNA binding domains and sequence elements within the mRNA with which they interact. However, despite substantial technological advancement in the ability to study RBPs and their target mRNA species, there are still considerable limitations in the approaches taken to study how *trans*‐acting proteins regulate translation. Thus there is significant scope for method development to allow a more uniform enrichment of the RBPome. Moreover, recent studies suggest that RBPs act in a combinatorial manner and thus it is important to know which combinations of proteins interact at specific sites within a given system and how these change upon cell perturbation. Currently this information can be captured, but generally in a low throughput manner. Coupling existing RBP capture methods with cell wide protein–protein crosslinking methods (Liu, Rijkers, Post, & Heck, 2015) may gain insight into which sets of proteins form regulatory complexes that interact with mRNA. Additionally, recent in silico analysis of canonical and novel RNA binding (Castello et al., 2016) has suggested enrichment of various PTMs such as tyrosine phosphorylation and lysine acetylation. Characterizing PTMs alongside RBP identification is necessary for a thorough understanding of the regulatory mechanisms that govern translation.

## CONFLICT OF INTEREST

The authors have declared no conflicts of interest for this article.
